# Cancer Screening Disparities Before and After the COVID-19 Pandemic

**DOI:** 10.1001/jamanetworkopen.2023.43796

**Published:** 2023-11-20

**Authors:** Aisha K. Lofters, Fangyun Wu, Eliot Frymire, Tara Kiran, Mandana Vahabi, Michael E. Green, Richard H. Glazier

**Affiliations:** 1 Peter Gilgan Centre for Women’s Cancers, Women’s College Hospital, Toronto, Ontario, Canada; 2 Department of Family and Community Medicine, University of Toronto, Toronto, Ontario, Canada; 3 ICES Central, Toronto, Ontario, Canada; 4 MAP Centre for Urban Health Solutions, St Michael’s Hospital, Toronto, Ontario, Canada; 5 Health Services and Policy Research Institute, Queen’s University, Kingston, Ontario, Canada; 6 ICES Queen’s, Kingston, Ontario, Canada; 7 Daphne Cockwell School of Nursing, Toronto Metropolitan University, Toronto, Ontario, Canada; 8 Department of Family Medicine, Queen’s University, Kingston, Ontario, Canada

## Abstract

**Importance:**

Breast, cervical, and colorectal cancer–screening disparities existed prior to the COVID-19 pandemic, and it is unclear whether those have changed since the pandemic.

**Objective:**

To assess whether changes in screening from before the pandemic to after the pandemic varied for immigrants and for people with limited income.

**Design, Setting, and Participants:**

This population-based, cross-sectional study, using data from March 31, 2019, and March 31, 2022, included adults in Ontario, Canada, the country’s most populous province, with more than 14 million people, almost 30% of whom are immigrants. At both dates, the screening-eligible population for each cancer type was assessed.

**Exposures:**

Neighborhood income quintile, immigrant status, and primary care model type.

**Main Outcomes and Measures:**

For each cancer screening type, the main outcome was whether the screening-eligible population was up to date on screening (a binary outcome) on March 31, 2019, and March 31, 2022. Up to date on screening was defined as having had a mammogram in the previous 2 years, a Papanicolaou test in the previous 3 years, and a fecal test in the previous 2 years or a flexible sigmoidoscopy or colonoscopy in the previous 10 years.

**Results:**

The overall cohort on March 31, 2019, included 1 666 943 women (100%) eligible for breast screening (mean [SD] age, 59.9 [5.1] years), 3 918 225 women (100%) eligible for cervical screening (mean [SD] age, 45.5 [13.2] years), and 3 886 345 people eligible for colorectal screening (51.4% female; mean [SD] age, 61.8 [6.4] years). The proportion of people up to date on screening in Ontario decreased for breast, cervical, and colorectal cancers, with the largest decrease for breast screening (from 61.1% before the pandemic to 51.7% [difference, −9.4 percentage points]) and the smallest decrease for colorectal screening (from 65.9% to 62.0% [difference, −3.9 percentage points]). Preexisting disparities in screening for people living in low-income neighborhoods and for immigrants widened for breast screening and colorectal screening. For breast screening, compared with income quintile 5 (highest), the β estimate for income quintile 1 (lowest) was −1.16 (95% CI, −1.56 to −0.77); for immigrant vs nonimmigrant, the β estimate was −1.51 (95% CI, −1.84 to −1.18). For colorectal screening, compared with income quintile 5, the β estimate for quntile 1 was −1.29 (95% CI, 16 −1.53 to −1.06); for immigrant vs nonimmigrant, the β estimate was −1.41 (95% CI, −1.61 to −1.21). The lowest screening rates both before and after the COVID-19 pandemic were for people who had no identifiable family physician (eg, moving from 11.3% in 2019 to 9.6% in 2022 up to date for breast cancer). In addition, patients of interprofessional, team-based primary care models had significantly smaller reductions in β estimates for breast (2.14 [95% CI, 1.79 to 2.49]), cervical (1.72 [95% CI, 1.46 to 1.98]), and colorectal (2.15 [95% CI, 1.95 to 2.36]) postpandemic screening and higher uptake of screening in general compared with patients of other primary care models.

**Conclusions and Relevance:**

In this cross-sectional study in Ontario that included 2 time points, widening disparities before compared with after the COVID-19 pandemic were found for breast cancer and colorectal cancer screening based on income and immigrant status, but smaller declines in disparities were found among patients of interprofessional, team-based primary care models than among their counterparts. Policy makers should investigate the value of prioritizing and investing in improving access to team-based primary care for people who are immigrants and/or with limited income.

## Key Points

**Question:**

Did preexisting disparities in cancer screening change after the COVID-19 pandemic?

**Findings:**

In this cross-sectional study of 9 471 513 adults in Ontario, Canada, who were eligible for breast, cervical, and colorectal cancer screening before the COVID-19 pandemic, assessed at 2 time points, preexisting disparities in screening for people living in lower-income neighborhoods and for immigrants significantly widened for both breast and colorectal cancer screening.

**Meaning:**

Results of this study suggest that policy makers should investigate the value of prioritizing and investing in improving access to team-based primary care for people who are immigrants and/or living with low income.

## Teaser

This cross-sectional study analyzes changes in breast, cervical, and colorectal cancer screening before and after the COVID-19 pandemic among adults in Ontario, Canada.

## Introduction

In March 2020, the World Health Organization declared a global pandemic of COVID-19.^1^ In response, cancer screening was paused in many jurisdictions around the world, including throughout North America, Europe, and Australia, as part of general health care system pauses of nonemergent care.^2,3,4,5,6,7^ After a decrease in the burden of COVID-19 cases over the next several months, jurisdictions began to gradually resume cancer screening.^6,7,8,9^ In the Canadian province of Ontario, this pause and gradual resumption led to a sizeable backlog in cancer screening. There were 41% fewer screening tests performed overall in Ontario in 2020 compared with 2019,^6,10^ and by December 2020, there was a backlog of more than 300 000 screening mammograms.^11^ The screening backlog was expected to be associated with delays in cancer diagnosis: between March and August 2020, there were, on average, 51% fewer high-grade cytological abnormalities detected through screening each month compared with 2019,^12^ and Tinmouth et al^13^ estimated that it would take 41 months to recover from the provincial backlog of colonoscopies, the recommended test after abnormal colorectal screening results with a fecal immunochemical test.

The COVID-19 cancer screening backlog was occurring in the context of longstanding provincial disparities in breast, cervical, and colorectal cancer screening for people who are immigrants or with limited income, despite 3 organized provincial screening programs that provide clear, evidence-based guidelines on screening eligibility, centralized correspondence to those due for screening, and family physician audit and feedback reports for patients in their practice.^14,15,16,17,18,19,20,21^ For example, a 2018 report from the provincial advisor on the quality of health care found that only 55% of women living in extremely income-limited, urban neighborhoods were up to date on cervical screening compared with 66% of women in the wealthiest, urban neighborhoods and that 45.6% of people living in extremely income-limited, urban neighborhoods were overdue for colorectal cancer screening compared with only 32.5% in the wealthiest, urban neighborhoods.^22^ Similarly, Vahabi et al^23^ found that 57% of immigrants were up to date on breast cancer screening compared with 66% of nonimmigrants.

It is unclear how the COVID-19 pandemic has affected preexisting screening disparities. Primary care physicians in Ontario were encouraged to consider prioritizing people who were underscreened or never screened for cancers when screening resumed,^24^ but it is also feasible that barriers to screening were heightened in the context of COVID-19 for people experiencing disparities. In this population-based, retrospective, cross-sectional study, we aimed to assess whether changes in screening from before the pandemic to March 2022 varied by income or immigration status.

## Methods

Research ethics board approval for this study was obtained by ICES (formerly known as the Institute for Clinical Evaluative Sciences) through the Privacy Impact Assessment of Sunnybrook Health Sciences Centre, Toronto, Ontario. ICES is an independent, nonprofit research institute, and its legal status under Ontario’s health information privacy law allows it to collect and analyze health care and demographic data, without consent, for health system evaluation and improvement. This study followed the Strengthening the Reporting of Observational Studies in Epidemiology (STROBE) reporting guideline.

### Setting

Ontario is Canada’s most populous province with more than 14 million people, of whom almost 30% are immigrants.^25^ Ontario has a universal health care system in which all Canadian citizens and permanent residents who live in the province can obtain medically necessary hospital and physician services, including cancer screening, at no cost through the Ontario Health Insurance Plan. There are more than 14 000 family physicians in Ontario, many of whom practice in patient enrollment models, in which patients formally enroll with a particular family physician who provides them with primary care.^26,27^ There are several types of patient enrollment models, including those in which physician payment is primarily fee for service, but there are also additional payments based on capitation and quality performance including cancer screening (referred to in this study as enhanced fee for service). The models with additional payments include those in which payment is primarily capitation based but with a small fee-for-service component and additional payments based on quality performance, including cancer screening (referred to in this study as capitation based, nonteam). Among primarily capitation-based models, those that include government-funded, interprofessional teams are known as family health teams. More than 77% of Ontario residents are formally enrolled with a family physician practicing in a patient enrollment model.^27,28^ There are also 101 community health centers in Ontario, which are nonprofit organizations that provide primary care and health-promotion services to patients and communities, with a focus on people experiencing marginalization, serving less than 2% of the population.^29^ These centers also are structured using interprofessional teams. Physicians working in community health centers are paid by salary.^30^ Family physicians who do not work in a patient enrollment model or in a community health center work under a traditional fee-for-service pay structure.

### Data Sources

We accessed several data sets available at ICES, including the Immigration, Refugees and Citizenship Canada Permanent Residents database, which consists of detailed demographic information on Ontario’s immigrants and refugees who arrived in the country beginning in 1985. Data include their country of birth, date of arrival, and immigrant category. The Postal Code Conversion File was used to obtain Rurality Index for Ontario (RIO) classification based on postal code of residence. The Registered Persons Database was used to determine residents’ health insurance coverage. We also accessed the Primary Care Population Database, which includes every Ontario resident who is eligible for primary care with active provincial health insurance coverage and a health care contact in the previous 7 to 11 years at a given point in time.^31^ This data set includes patient demographic characteristics, family physician enrollment status, health care usage information, and cancer screening status. The Ontario Health Insurance Plan claims database and Discharge Abstract Database were used for billing code and diagnostic information and for exclusions. The ICES Physician Database and Corporate Provider Database were used to obtain information on family physicians. The Canadian 2016 Census of Population was used to determine neighborhood income quintile (given as Q1-Q5) based on postal code of residence. These data sets were linked using unique encoded identifiers and analyzed at ICES using SAS, version 8.3 (SAS Institute Inc).

### Study Outcome

For each cancer screening type, we assessed whether the screening-eligible population was up to date on screening (a binary outcome) at 2 time points: March 31, 2019 (prior to the COVID-19 pandemic), and March 31, 2022. Being up to date on screening was defined as having had a mammogram in the previous 2 years, a Papanicolaou test in the previous 3 years, and a fecal test in the previous 2 years or a flexible sigmoidoscopy or colonoscopy in the previous 10 years.^32^

### Defining Screening-Eligible Populations

At each of the 2 time points, we defined the screening-eligible population for each cancer type. We started with all residents of Ontario who had at least 5 years’ history of Ontario Health Insurance Plan coverage on the index dates (March 31, 2019, and March 31, 2022). Women eligible for breast screening were defined as those aged 52 to 69 years, excluding those with a history of breast cancer or mastectomy. Women eligible for cervical screening were defined as those aged 23 to 69 years, excluding those with a history of cervical cancer or hysterectomy. People eligible for colorectal screening were defined as those aged 52 to 74 years, excluding those who had a history of colorectal cancer or inflammatory bowel diseases.^31^ The lower age thresholds were chosen to allow for individuals to be eligible for screening for the full look-back window.

### Study Variables

We described each study cohort based on age category, sex, neighborhood income quintile, immigrant status (immigrant or not), primary care model, rurality of residence based on postal code using the RIO classification,^33^ and health care use over the prior 2 years using resource utilization bands (RUBs) (ranges from 0 to 5, with higher values indicating a very high user of health care services) from the case-mix Johns Hopkins ACG System, version 10.0 (Johns Hopkins Medicine),^34^ which uses outpatient billing and inpatient hospital records. For immigrants, we also determined their world region of origin based on country of birth and a previously published modification of the World Bank classification system, as screening status has been shown to vary by region of origin.^35,36^

### Statistical Analysis

We used counts and frequencies to describe the 3 screening-eligible cohorts based on the aforementioned variables as of March 31, 2019. We assessed up-to-date status for each screening type at 6-month intervals, from April 1, 2012, to April 1, 2022, for the overall cohorts, as well as stratified by income quintile and immigrant status. We also compared breast, cervical, and colorectal screening at our 2 time points for the overall cohorts as well as stratified by our study variables.

For each screening type, we conducted multivariable regression analyses using generalized linear models, with the rate difference between the postpandemic and prepandemic time points as the outcome. To assess the rate difference after and before the pandemic, we aggregated individual-level data to unique combinations of each category of age group, sex (for colorectal cancer only), income quintile, immigration status, patient enrollment model, RIO categories, and RUBs, all of which were included in the model. The mean number of eligible people from before and after the pandemic in each cluster was used as weight to account for different size of denominators. To avoid small denominators and to prevent unstable rates in the regression models, we combined family health teams and community health centers into 1 category, as both use interprofessional teams. We combined those in RUBs 0 and 1 and 4 and 5, and we excluded people with no identifiable family physician. To reduce the outcome of unstable results associated with small clusters, we excluded clusters having fewer than 50 eligible people either before or after the pandemic in the generalized linear models. This excluded no more than 0.36% (for breast screening) of the 2019 population and no more than 0.46% (for breast screening) of the 2022 population. The threshold for statistical significance was 2-sided *P* < .05.

## Results

On March 31, 2019, there were 1 666 943 women eligible for breast screening (mean [SD] age, 59.9 [5.1] years), 3 918 225 women eligible for cervical screening (mean [SD] age, 45.5 [13.2] years), and 3 886 345 people eligible for colorectal screening (51.4% female and 48.6% male; mean [SD] age, 61.8 [6.4] years) in Ontario (Table 1). Between 26.1% and 28.4% of people were enrolled in a family health team, less than 1% with community health centers, and between 3.7% and 4.9% with no identifiable family physician. East Asia and the Pacific, Europe and Central Asia, and South Asia were the most common source regions for immigrant Ontario residents.

**Table 1.** Characteristics of Ontario Residents Eligible for Breast, Cervical, and Colorectal Cancer Screening on March 31, 2019

Variable	Individuals, No. (%)		
	Breast (n = 1 666 943)	Cervical (n = 3 918 225)	Colorectal (n = 3 886 345)
Sex			
Female	1 666 943 (100)	3 918 225 (100)	1 996 166 (51.4)
Male	0	0	1 890 179 (48.6)
Age at index, y			
Mean (SD)	59.9 (5.1)	45.5 (13.2)	61.8 (6.4)
Median (IQR)	60 (56-64)	46 (34-57)	61 (56-67)
Age category at index, y			
21-34	0	1 009 644 (25.8)	0
35-49	0	1 289 644 (32.9)	0
50-69	1 666 943 (100)	1 618 937 (41.3)	3 271 389 (84.2)
70-74	0	0	614 956 (15.8)
Neighborhood income quintile			
1 (Lowest)	305 675 (18.3)	744 403 (19.0)	713 961 (18.4)
2	328 509 (19.7)	768 806 (19.6)	763 896 (19.7)
3	334 859 (20.1)	793 414 (20.2)	781 048 (20.1)
4	334 497 (20.1)	800 571 (20.4)	779 234 (20.1)
5 (Highest)	361 467 (21.7)	806 052 (20.6)	842 352 (21.7)
Missing	1936 (0.1)	4979 (0.1)	5854 (0.2)
Model of primary care			
Capitation based (nonteam)	562 510 (33.7)	1 244 806 (31.8)	1 292 696 (33.3)
Enhanced fee for service	478 900 (28.7)	1 237 524 (31.6)	1 108 047 (28.5)
Community health centers	14 763 (0.9)	26 685 (0.7)	34 007 (0.9)
Family health teams	472 934 (28.4)	1 021 294 (26.1)	1 082 093 (27.8)
Traditional fee for service	76 111 (4.6)	196 425 (5.0)	188 233 (4.8)
No family physician	61 725 (3.7)	191 491 (4.9)	181 269 (4.7)
Rurality			
Large urban	670 823 (40.2)	1 693 154 (43.2)	1 549 802 (39.9)
Urban	473 287 (28.4)	1 185 463 (30.3)	1 093 161 (28.1)
Small urban	358 554 (21.5)	731 450 (18.7)	844 749 (21.7)
Rural	164 279 (9.9)	308 158 (7.9)	398 633 (10.3)
Resource utilization band^a^			
0 (Nonuser)	100 803 (6.0)	328 710 (8.4)	280 404 (7.2)
1	46 539 (2.8)	160 983 (4.1)	109 580 (2.8)
2	203 783 (12.2)	591 843 (15.1)	481 450 (12.4)
3	963 798 (57.8)	2 022 085 (51.6)	2 155 620 (55.5)
4	247 309 (14.8)	685 720 (17.5)	572 804 (14.7)
5 (Highest user)	104 711 (6.3)	128 884 (3.3)	286 487 (7.4)
World region of birth			
Canada	1 357 766 (81.5)	3 001 057 (76.6)	3 193 633 (82.2)
East Asia and the Pacific	95 667 (5.7)	265 591 (6.8)	192 576 (5.0)
Europe and Central Asia	62 868 (3.8)	159 271 (4.1)	143 021 (3.7)
Latin America and the Caribbean	45 813 (2.7)	123 177 (3.1)	98 802 (2.5)
Middle East and North Africa	23 034 (1.4)	81 453 (2.1)	58 820 (1.5)
North America	6105 (0.4)	17 084 (0.4)	12 151 (0.3)
South Asia	59 433 (3.6)	215 030 (5.5)	148 524 (3.8)
Sub-Saharan Africa	15 880 (1.0)	54 754 (1.4)	37 907 (1.0)
Unknown	377 (<1.0)	808 (<1.0)	911 (<1.0)

^a^ Resource utilization bands range from 0 to 5, with higher values indicating a very high user of health care services. In this study, a nonuser is 0, and the highest user is 5.

**Figure 1.**
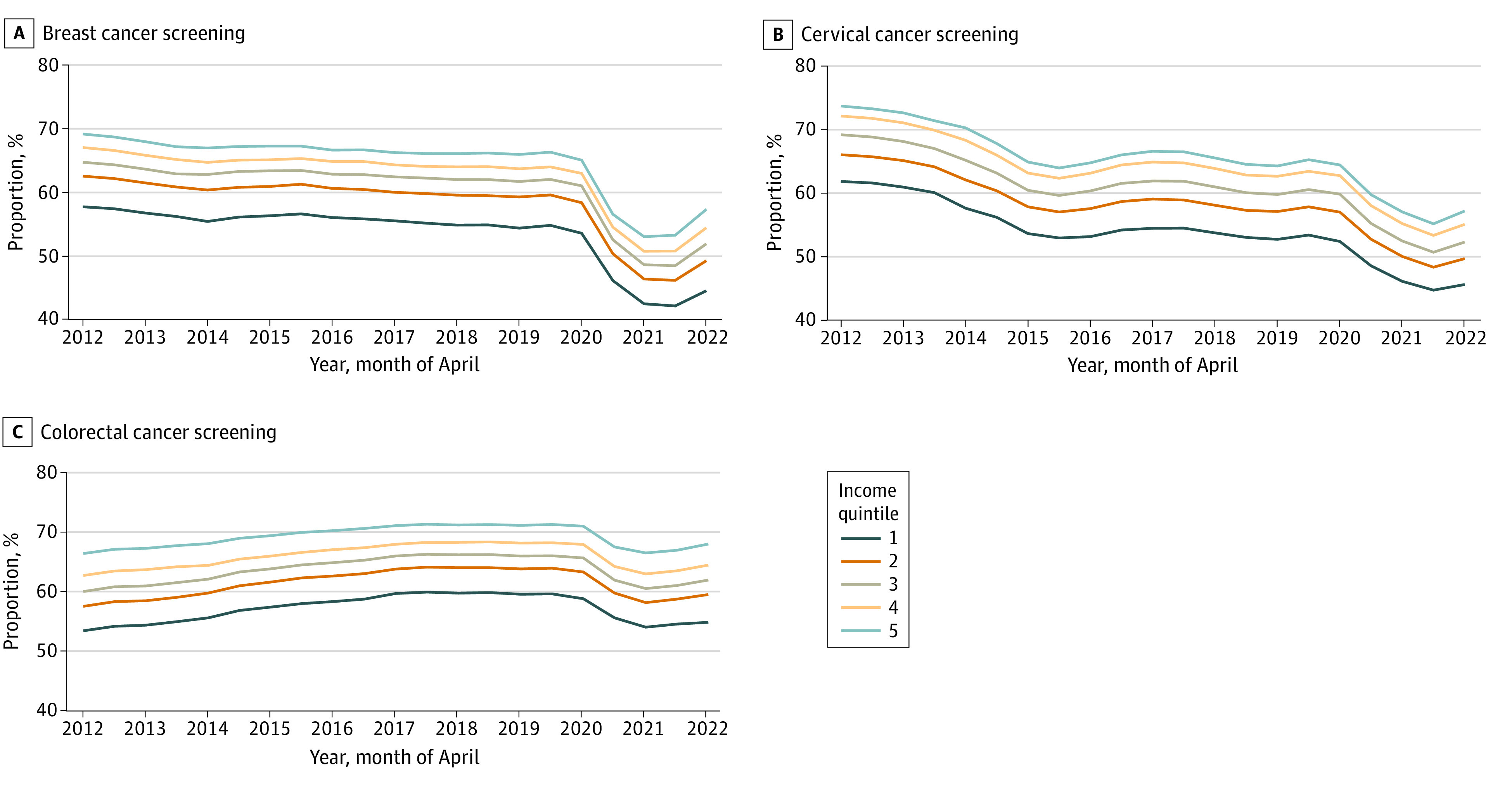
Percentage of Ontario Residents Eligible and Up to Date on Breast, Cervical, and Colorectal Cancer Screening at 6-Month Intervals, From 2012 to 2022, Stratified by Neighborhood Income Quintile Income quintile 1 represents the lowest; quintile 5, the highest.

**Figure 2.**
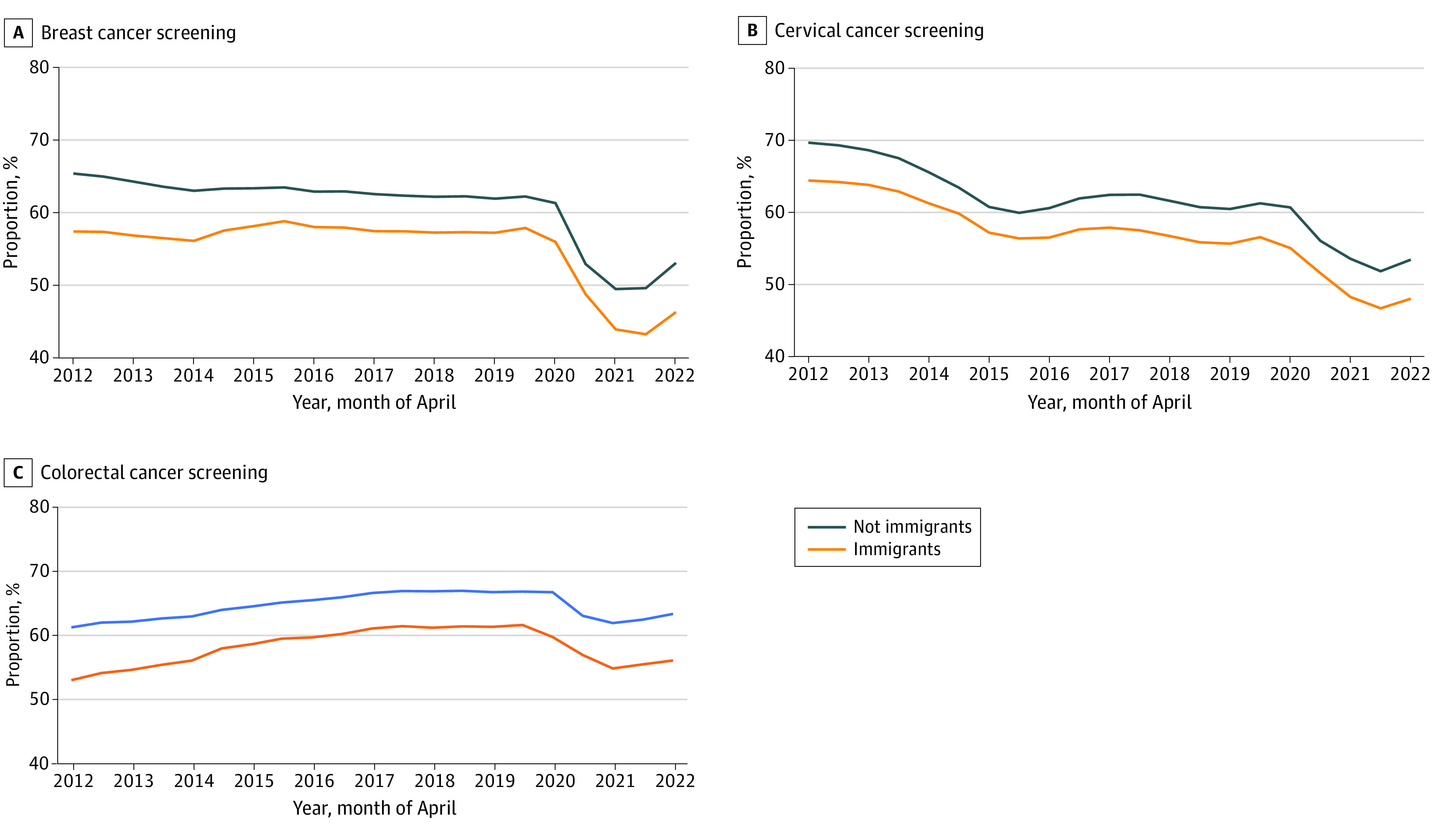
Percentage of Ontario Residents Eligible and Up to Date on Breast, Cervical, and Colorectal Cancer Screening at 6-Month Intervals, From 2012 to 2022, Stratified by Immigrant Status

Figure 1 shows the cancer screening status from 2012 to 2022, stratified by income quintile and by each cancer screening type. A clear and sustained income gradient of approximately 12% to 13% was observed over time for all screening types. Prior to the pandemic, breast screening up-to-date status had been relatively steady, cervical screening up-to-date status had been decreasing, and colorectal screening up-to-date status had been gradually increasing. All screening types decreased as of spring 2020 (most notably breast cancer screening, for which screening for those in Q1 declined from 53.6% in April 2020 to 42.5% in April 2021), with a gradual increase as of fall 2021 vs 56.3% to 53.0% in Q5. Figure 2 shows similar results, stratified by immigrant status. Immigrants tended to have a consistently lower up-to-date screening status vs nonimmigrants over time (eg, a 5.3% difference in cervical screening in April 2012 and a 5.4% difference in April 2021).

From March 31, 2019 (before the pandemic), to March 31, 2022, the proportion of people up to date for each screening type declined, by 9.4 percentage points for breast (61.1% to 51.7%), 7.3 percentage points for cervical (59.4% to 52.1%), and 3.9 percentage points for colorectal (65.9% to 62.0%) cancer screening (Table 2). For breast screening, women in Q5 had a smaller decrease (8.6 percentage points) than women in other income quintiles (9.2 percentage points to 10.0 percentage points), as did women enrolled in family health teams (8.1 percentage points) vs those in other primary care model types (8.6 percentage points to 10.5 percentage points), excluding those with no family physician. Both groups also had the highest screening uptake prior to the pandemic. For cervical screening, women enrolled in family health teams had the smallest reduction (6.4 percentage points) between the 2 periods, excluding those with no family physician, although women using community health centers had the highest screening uptake among primary care model types in both time periods (69.7% and 62.7%). The highest users of the health care system only had a 2.5 percentage point decrease in cervical screening. For colorectal screening, among primary care model types, both the smallest decrease and highest screening rate after the pandemic were seen in those enrolled in family health teams (2.3 percentage points [70.1% to 67.7%]). Those with the highest health care use had a similar pattern (1.9 percentage point decrease [74.2% to 72.4%]). For all 3 screening types, people with no family physician had markedly lower screening rates and a negligible difference between the 2 time periods, moving from 11.3% in 2019 to 9.6% up to date in 2022 for breast cancer, 10.8% to 10.2% for cervical cancer, and 17.6% to 16.8% for colorectal cancer. Furthermore, for all 3 screening types, immigrants from East Asia and the Pacific had the largest pandemic screening reductions but notably did not have the lowest postpandemic screening rates.

**Table 2.** Individuals Up to Date on Cancer Screening on the Study Dates for Overall Study Cohorts Stratified by Study Variables

Variable	Breast			Cervical			Colorectal		
	Before the pandemic (n = 1 666 943), No. (%)	After the pandemic (n = 1 689 049), No. (%)	Difference, percentage points	Before the pandemic (n = 3 918 225), No. (%)	After the pandemic (n = 3 946 553), No. (%)	Difference, percentage points	Before the pandemic (n = 3 886 345), No. (%)	After the pandemic (n = 4 022 709), No. (%)	Difference, percentage points
Total	1 019 239 (61.1)	873 739 (51.7)	−9.4	2 325 799 (59.4)	2 054 958 (52.1)	−7.3	2 562 618 (65.9)	2 494 589 (62.0)	−3.9
Sex									
Female	1 019 239 (61.1)	873 739 (51.7)	−9.4	2 325 799 (59.4)	2 054 958 (52.1)	−7.3	1 363 466 (68.3)	1 335 342 (64.6)	−3.7
Male	NA	NA	NA	NA	NA	NA	1 199 152 (63.4)	1 159 247 (59.3)	−4.2
Age category at index, y									
21-34	NA	NA	NA	561 738 (55.6)	480 047 (48.1)	−7.6	NA	NA	NA
35-49	NA	NA	NA	799 383 (62.0)	708 051 (54.9)	−7.1	NA	NA	NA
50-69	1 019 239 (61.1)	873 739 (51.7)	−9.4	964 678 (59.6)	866 860 (52.3)	−7.3	2 114 274 (64.6)	2 031 894 (60.6)	−4.0
70-74	NA	NA	NA	NA	NA	NA	448 344 (72.9)	462 695 (68.9)	−4.0
Neighborhood income quintile									
1 (Lowest)	166 155 (54.4)	136 626 (44.6)	−9.8	392 118 (52.7)	336 288 (45.6)	−7.1	425 384 (59.6)	401 150 (54.9)	−4.7
2	194 588 (59.2)	161 453 (49.3)	−10.0	438 597 (57.0)	380 430 (49.6)	−7.4	487 627 (63.8)	463 860 (59.5)	−4.3
3	206 465 (61.7)	175 523 (51.9)	−9.8	473 888 (59.7)	417 529 (52.2)	−7.5	515 465 (66.0)	499 108 (62.0)	−4.0
4	212 868 (63.6)	187 511 (54.4)	−9.2	501 086 (62.6)	450 019 (55.0)	−7.6	531 338 (68.2)	528 010 (64.5)	−3.7
5 (Highest)	238 145 (65.9)	210 980 (57.3)	−8.6	517 622 (64.2)	466 757 (57.1)	−7.1	599 451 (71.2)	596 963 (68.0)	−3.1
Missing	1018 (52.6)	1646 (46.5)	−6.1	2488 (50.0)	4025 (44.3)	−5.6	3353 (57.3)	5498 (53.9)	−3.4
Model of primary care									
Capitation based (nonteam)	364 074 (64.7)	325 486 (55.5)	−9.2	797 176 (64.0)	742 072 (57.2)	−6.9	908 421 (70.3)	923 351 (67.2)	−3.1
Enhanced fee for service	289 908 (60.5)	225 608 (50.0)	−10.5	717 976 (58.0)	575 893 (50.0)	−8.0	738 870 (66.7)	658 708 (61.8)	−4.9
Community health centers	9164 (62.1)	9966 (53.3)	−8.7	18 605 (69.7)	22 125 (62.7)	−7.0	23 435 (68.9)	27 994 (64.1)	−4.8
Family health teams	311 214 (65.8)	273 051 (57.7)	−8.1	675 216 (66.1)	604 553 (59.8)	−6.4	758 373 (70.1)	747 743 (67.7)	−2.3
Traditional fee for service	37 881 (49.8)	31 673 (41.2)	−8.6	96 185 (49.0)	85 019 (41.9)	−7.1	101 601 (54.0)	95 791 (50.3)	−3.6
No family physician	6998 (11.3)	7955 (9.6)	−1.7	20 641 (10.8)	25 296 (10.2)	−0.6	31 918 (17.6)	41 002 (16.8)	−0.8
Rurality									
Large urban	401 981 (59.9)	339 459 (50.3)	−9.7	977 717 (57.7)	849 609 (50.4)	−7.3	1 006 033 (64.9)	970 892 (60.8)	−4.2
Urban	295 337 (62.4)	255 462 (52.6)	−9.8	715 392 (60.3)	631 059 (52.5)	−7.9	738 563 (67.6)	720 757 (62.9)	−4.6
Small urban	223 202 (62.3)	193 965 (53.7)	−8.6	451 182 (61.7)	409 496 (55.0)	−6.7	562 210 (66.6)	551 260 (63.5)	−3.0
Rural	98 719 (60.1)	84 853 (50.9)	−9.2	181 508 (58.9)	164 794 (52.5)	−6.4	255 812 (64.2)	251 680 (61.1)	−3.0
Resource utilization band^a^									
0 (Nonuser)	12 679 (12.6)	20 265 (12.9)	0.3	41 431 (12.6)	62 899 (13.5)	0.9	54 413 (19.4)	95 714 (22.2)	2.8
1	20 484 (44.0)	18 744 (39.4)	−4.6	76 023 (47.2)	61 238 (43.2)	−4.0	51 631 (47.1)	55 550 (49.8)	2.7
2	109 745 (53.9)	105 414 (45.3)	−8.5	327 657 (55.4)	296 011 (47.7)	−7.7	271 438 (56.4)	300 031 (54.4)	−2.0
3	638 270 (66.2)	529 197 (57.7)	−8.5	1 325 191 (65.5)	1 117 544 (58.7)	−6.8	1 534 202 (71.2)	1 429 114 (68.3)	−2.8
4	171 676 (69.4)	139 640 (61.4)	−8.0	479 333 (69.9)	435 152 (65.0)	−4.9	438 306 (76.5)	399 780 (74.0)	−2.5
5 (Highest user)	66 385 (63.4)	60 479 (56.3)	−7.1	76 164 (59.1)	82 114 (56.6)	−2.5	212 628 (74.2)	214 400 (72.4)	−1.9
World region of birth									
Canada	841 975 (62.0)	706 276 (53.2)	−8.8	1 815 332 (60.5)	1 582 083 (53.4)	−7.1	2 136 817 (66.9)	2 033 826 (63.5)	−3.4
East Asia and the Pacific	57 992 (60.6)	53 253 (47.3)	−13.4	152 117 (57.3)	135 561 (48.0)	−9.3	128 619 (66.8)	138 460 (60.4)	−6.4
Europe and Central Asia	32 859 (52.3)	30 595 (43.7)	−8.6	93 304 (58.6)	82 288 (51.1)	−7.5	85 161 (59.5)	91 234 (56.1)	−3.4
Latin America and the Caribbean	28 929 (63.1)	28 042 (53.3)	−9.8	75 598 (61.4)	69 669 (54.8)	−6.6	64 420 (65.2)	69 046 (59.6)	−5.7
Middle East and North Africa	13 595 (59.0)	14 006 (49.0)	−10.0	41 727 (51.2)	44 277 (45.7)	−5.6	35 431 (60.2)	41 766 (56.6)	−3.6
North America	3202 (52.4)	3084 (46.1)	−6.4	8773 (51.4)	8469 (45.4)	−6.0	7510 (61.8)	8266 (59.9)	−1.9
South Asia	32 104 (54.0)	29 815 (42.6)	−11.4	109 955 (51.1)	104 788 (44.0)	−7.2	81 918 (55.2)	87 044 (49.1)	−6.0
Sub-Saharan Africa	8339 (52.5)	8474 (42.8)	−9.7	28 542 (52.1)	27 410 (45.9)	−6.3	22 146 (58.4)	24 340 (52.2)	−6.2
Unknown	244 (64.7)	194 (48.6)	−16.1	451 (55.8)	413 (47.8)	−8.0	596 (65.4)	607 (60.3)	−5.1

Abbreviation: NA, not applicable.

^a^ Resource utilization bands range from 0 to 5, with higher values indicating a very high user of health care services. In this study, a nonuser was 0, and the highest user was 5.

Multiple regression analysis results for those with a family physician are shown in Table 3. For breast screening, older age (β estimate, −1.02 [95% CI, −1.26 to −0.77] for age 60 to 69 years vs 50 to 69 years), lower neighborhood income quintile (compared with Q5, β estimate for Q1: −1.16 [95% CI, −1.56 to −0.77]; for Q2: −1.15 [95% CI, −1.54 to −0.77]; for Q3: −1.01 [95% CI, −1.39 to −0.63]; for Q4: −0.44 [95% CI, −0.82 to −0.06]); and immigrant status (β estimate, −1.51 [95% CI, −1.84 to −1.18]) for immigrant vs nonimmigrant) were independently significantly associated with larger reductions in screening after the pandemic. For cervical screening, differences based on income (compared with Q5, β estimate for Q1: −0.10 [95% CI, −0.40 to 0.20]; for Q2: −0.23 [95% CI, −0.53 to 0.06]; for Q3 −0.30 [95% CI, −0.59 to −0.01]; and for Q4: −0.34 [95% CI, −0.63 to −0.05]) and on immigrant status (β estimate, −0.13 [95% CI, −0.36 to 0.11] for immigrant vs nonimmigrant) were small and/or not significant. For colorectal screening, female sex was associated with a slightly smaller pandemic reduction in screening (β estimate, 0.27 [95% CI, 0.12-0.41]) vs male sex, and lower income quintile (compared with Q5, β estimate for Q1: −1.29 [95% CI, −1.53 to −1.06]; for Q2: −0.81 [95% CI, −1.04 to −0.58]; for Q3: −0.64 [95% CI, −0.86 to −0.41]; and for Q4: −0.37 [95% CI, −0.59 to −0.14]) and immigrant status (β estimate, −1.41; 95% CI, −1.61 to −1.21 for immigrant vs nonimmigrant) were associated with larger reductions in screening after the pandemic. Across all screening types, interprofessional teams (family health teams and community health centers; β estimate for breast screening: 2.14 [95% CI, 1.79 to 2.49]; for cervical screening, 1.72 [95% CI, 1.46 to 1.98], and for colorectal screening, 2.15 [95% CI, 1.95 to 2.36]), followed by nonteam capitation payment models (β estimate for breast screening: 1.34 [95% CI, 1.02 to 1.65]; for cervical screening, 1.40 [95% CI, 1.17 to 1.64], and for colorectal screening, 1.86 [95% CI, 1.67 to 2.05]), were significantly associated with the smallest pandemic reductions in screening after the pandemic.

**Table 3.** Multivariable Regression Analyses for Cancer Screening Type With the Rate Difference Between the Study Dates as the Outcome, Adjusting for Variables^a^

Variable	Breast, β estimate (95% CI)^b^	*P* value	Cervical, β estimate (95% CI)^b^	*P* value	Colorectal, β estimate (95% CI)^b^	*P* value
Sex						
Female	NA	NA	NA	NA	0.27 (0.12 to 0.41)	<.001
Male	NA	NA	NA	NA	1 [Reference]	NA
Age category, y						
50-69 vs 21-49	NA	NA	0.26 (0.07 to 0.45)	.01	NA	NA
60-69 vs 50-59	−1.02 (−1.26 to −0.77)	<.001	NA	NA	NA	NA
70-74 vs 50-69	NA	NA	NA	NA	−0.12 (−0.32 to 0.08)	.24
Neighborhood income quintile						
1(Lowest)	−1.16 (−1.56 to −0.77)	<.001	−0.10 (−0.40 to 0.20)	.51	−1.29 (−1.53 to −1.06)	<.001
2	−1.15 (−1.54 to −0.77)	<.001	−0.23 (−0.53 to 0.06)	.13	−0.81 (−1.04 to −0.58)	<.001
3	−1.01 (−1.39 to −0.63)	<.001	−0.30 (−0.59 to −0.01)	.04	−0.64 (−0.86 to −0.41)	<.001
4	−0.44 (−0.82 to −0.06)	.02	−0.34 (−0.63 to −0.05)	.02	−0.37 (−0.59 to −0.14)	.001
5 (Highest)	1 [Reference]	NA	1 [Reference]	NA	1 [Reference]	NA
Immigrant status						
Nonimmigrant	1 [Reference]	NA	1 [Reference]	NA	1 [Reference]	NA
Immigrant	−1.51 (−1.84 to −1.18)	<.001	−0.13 (−0.36 to 0.11)	.29	−1.41 (−1.61 to −1.21	<.001
Model of primary care						
Enhanced fee for service	1 [Reference]	NA	1 [Reference]	NA	1 [Reference]	NA
Capitation based (nonteam)	1.34 (1.02 to 1.65)	<.001	1.40 (1.17 to 1.64)	<.001	1.86 (1.67 to 2.05)	<.001
Traditional fee for service	0.92 (0.30 to 1.54)	.004	0.13 (−0.30 to 0.57)	.55	0.24 (−0.11 to 0.60)	.18
Community health centers and family health teams	2.14 (1.79 to 2.49)	<.001	1.72 (1.46 to 1.98)	<.001	2.15 (1.95 to 2.36)	<.001
Rurality						
Large urban	1 [Reference]	NA	1 [Reference]	NA	1 [Reference]	NA
Urban	−0.38 (−0.68 to −0.08)	.01	−0.28 (−0.50 to −0.05)	.02	−0.72 (−0.90 to −0.54)	<.001
Small urban	−0.44 (−0.79 to −0.09)	.01	0.04 (−0.23 to 0.31)	.79	−0.14 (−0.35 to 0.06)	.18
Rural	−1.24 (−1.71 to −0.78)	<.001	0.21 (−0.17 to 0.59)	.28	−0.05 (−0.32 to 0.22)	.72
Resource utilization band^c^						
0 and 1	3.14 (2.66 to 3.62)	<.001	1.78 (1.45 to 2.10)	<.001	3.25 (2.97 to 3.52)	<.001
2	−0.72 (−1.10 to −0.34)	<.001	−1.14 (−1.41 to −0.88)	<.001	0.22 (−0.01 to 0.44)	.06
3	1 [Reference]	NA	1 [Reference]	NA	1 [Reference]	NA
4 and 5	0.76 (0.44 to 1.07)	<.001	2.10 (1.86 to 2.33)	<.001	0.61 (0.42 to 0.79)	<.001

Abbreviation: NA, not applicable.

^a^ Generalized linear models were used for multivariable regression analyses.

^b^ The β estimate represents the percentage change in the rate difference vs the reference group, with negative values representing a larger decrease in screening after the pandemic and positive values representing a smaller decrease in screening after the pandemic.

^c^ Resource utilization bands range from 0 to 5, with higher values indicating a very high user of health care services. In the present study, a nonuser is 0, and the highest user is 5.

## Discussion

In this population-based cross-sectional study comparing cancer screening prior to the COVID-19 pandemic vs in the postpandemic period, we found that the proportion of people up to date on screening in Ontario decreased for breast, cervical, and colorectal cancers, with the largest decrease for breast screening (9.4 percentage points) and the smallest decrease for colorectal screening (3.9 percentage points). Preexisting disparities in screening for people living in low-income neighborhoods and for immigrants widened for both breast and colorectal screening. The lowest screening rates both before and after the pandemic were for people who had no identifiable family physician, in which rates were no higher than 17.6% (colorectal screening in 2019) and were as low as 9.6% (breast screening in 2022). We also found that patients of interprofessional models, such as family health teams and community health centers, had significantly smaller reductions in postpandemic screening and higher screening rates at each time point compared with other primary care patients.

Our findings suggest that access to primary care, as well as the type of primary care model to which one has access, played a crucial role in cancer-screening recovery after the COVID-19 pandemic. These results may make a compelling argument for expanding access to interprofessional, team-based primary care as a method of increasing cancer-screening uptake (and other quality of care^37^) province-wide. Among interprofessional teams, nonphysician practitioners may play a role in identifying patients who are overdue for screening and who need screening outreach or screening education and in performing and/or ordering screening tests. However, efforts to expand access to team-based care must center on health equity (ie, ensuring that immigrants and/or people with limited income are prioritized for team-based primary care).^37,38,39^ These efforts will require significant investment: although community health centers are designed for people experiencing marginalization, they currently serve less than 2% of the province’s population, and family health teams have traditionally been least available in geographic areas with the greatest need.^40^

Our results are in line with other literature. Several US studies have shown sociodemographic variation in COVID-associated decreases in cancer screening, with the largest reductions in breast cancer screening associated with African American and Asian women and women of other races and women who were insured by Medicaid.^41,42,43^ Fedewa et al^44^ found that US residents who became unemployed during the pandemic were 10% to 30% less likely to be screened for cancers than employed adults. In their analysis of electronic medical records from over 40 000 primary care patients in Michigan, Gorin et al^45^ observed an abrupt decrease in cancer screening between March and June 2020 but with a more modest decrease for fecal immunochemical tests.

### Limitations

This study has several limitations. First, we limited our population to Ontario residents who had at least 5 years of health insurance plan coverage on our index dates, but a screening colonoscopy is only required once every 10 years if results are normal; thus, we may have categorized people who had screening colonoscopies in other jurisdictions before moving to Ontario as unscreened. However, a requirement of 10 years of health plan coverage would have eliminated a large number of people from the study. Second, available data did not allow us to identify those who may have been at increased risk of cancer (eg, due to family history or previous abnormal test results). Provincial screening guidelines are different for individuals at increased risk.^32^ Third, people who immigrated to Ontario prior to 1985 or lived in another province before moving to Ontario would not have been included in the Immigration, Refugees and Citizenship Canada Permanent Residents database and would have been inadvertently classified as nonimmigrants. However, this would likely bias our results to the null. Fourth, neighborhood income is not an individual-level variable and may have subjected our results to ecological fallacy. This likely would also have biased our results to the null. Finally, to avoid small denominators and to prevent unstable rates in our regression models, we combined some groups and excluded people with no family physician. This reduced the information that the model provided, but the regrouping was based on the relevance and nature of the data, and we deemed it the most appropriate approach.

## Conclusions

This population-based, cross-sectional study in Ontario, Canada, found that the COVID-19 pandemic was associated with a decrease in up-to-date status for breast, cervical, and colorectal cancer screening and that people with limited income and immigrants generally had larger decreases than their counterparts. We also found that people without family physicians had very low screening uptake both before and after the pandemic and that, among those with family physicians, access to interprofessional, team-based primary care may have been protective for screening during the pandemic. Policy makers should investigate the value of prioritizing and investing in improving access to team-based primary care for people who are immigrants and/or with limited income.

## Notes

Supplement.
Data Sharing Statement
