# Supplementary material for: Cancer Screening Disparities Before and After the COVID-19 Pandemic
Source: JAMA Netw Open. 2023 Nov 20;6(11):e2343796. doi: 10.1001/jamanetworkopen.2023.43796 (PMC10660460; doi:10.1001/jamanetworkopen.2023.43796)
Supplement: Supplement. — Data Sharing Statement [file jamanetwopen-e2343796-s001.pdf]

Data Sharing Statement

Lofters. Cancer Screening Disparities Before and After the COVID-19 Pandemic. *JAMA Netw Open*. Published November 20, 2023. doi:10.1001/jamanetworkopen.2023.43796

Data
Data available: No

Additional Information
Explanation for why data not available: The data sets from this study are held securely in coded form at ICES. Data-sharing agreements prohibit ICES from making the data sets publicly available, but access may be granted to those who meet pre-specified criteria for confidential access, available at www.ices.on.ca/DAS. The complete data set creation plan, and underlying analytic code are available from the authors upon request, understanding that the programs may rely upon coding templates or macros unique to ICES.
